# Influence of cathode materials on thermal characteristics of lithium-ion batteries

**DOI:** 10.3389/fchem.2024.1324840

**Published:** 2024-03-12

**Authors:** Yuan Yuan, Qian Ma, Xiangqian Zhang, Fan Zhang, Xiangning Song, Hongchuan Xin, Guiru Zhu, Hongzhe Zhang

**Affiliations:** ^1^ State Key Laboratory of Safety and Control for Chemicals, SINOPEC Research Institute of Safety Engineering Co., Ltd, Qingdao, China; ^2^ National Registration Center for Chemicals, Ministry of Emergency Management of the People’s Republic of China, Qingdao, China; ^3^ Key Laboratory of Biofuels, Qingdao Institute of Bioenergy and Bioprocess Technology, Chinese Academy of Science, Qingdao, China; ^4^ Key Laboratory of Marine Chemistry Theory and Technology, Ministry of Education, College of Chemistry and Chemical Engineering, Ocean University of China, Qingdao, China

**Keywords:** lithium-ion battery, cathode material, calorimetry, thermal runaway, isothermal condition

## Abstract

In this work, the thermal stability of four types of 18,650 lithium-ion batteries with LiCoO_2_ (LCO), LiFePO_4_ (LFP), LiNi_0.8_Co_0.1_Mn_0.1_O_2_ (NCM811) and LiNi_0.8_Co_0.15_Al_0.05_O_2_ (NCA) materials as cathodes are experimentally investigated by the accelerating rate calorimeter (ARC) and the isothermal battery testing calorimeter (iso-BTC) under adiabatic and isothermal conditions, respectively. The thermal runaway danger level of these batteries can be ranked as LCO > NCA > NCM811 >> LFP by judging from the values of T_max_ and HR_max, nominal_. The higher the nickel and cobalt content, the higher the lithium-ion battery capacity, but the worse the thermal stability. The Q_total_ of NCA is the largest in the complete standard charge and discharge process, due to that the capacity of NCA is significantly higher than that of the other three batteries, resulting in remarkable increase in Q_irre_ proportioned to the square of the current. When the ambient temperature rises, the energy release decreases owing to the decrease in the internal resistance of the battery. These studies are expected to have important implications for the subsequent safe design of commercial lithium-ion batteries with different cathode materials.

## 1 Introduction

In recent years, as ambient protection has received more and more attention, energy conservation, emission reduction and energy structure transformation have become international trends (Zhu et al., 2019; Liu et al., 2022). At the same time, lithium-ion batteries, as an energy carrier that can realize the mutual conversion of electric energy and chemical energy, are considered to be the best solution for the new energy vehicle and power battery industries (Amine et al., 2010; Zheng et al., 2013; Chen et al., 2014; Huang et al., 2021). However, with the continuous occurrence of combustion and explosion accidents (Wang et al., 2012; Bugryniec et al., 2023), the safety of lithium-ion batteries is a key issue for their further development (Zheng et al., 2013; Larsson et al., 2016; Willstrand et al., 2023). One of the key elements of battery safety is the cathode material, which also affects battery performance, cycle life and manufacturing cost. Currently, the most widely used cathode materials include LiCoO_2_ (LCO), LiFePO_4_ (LFP) and LiNi_x_Co_y_Mn_1-x-y_O_2_ (NCM) ternary materials (Wang et al., 2023).

In 1980, Mizushima et al. first employed LCO as a cathode material for lithium-ion batteries, and obtained an operating voltage of over 4 V and good reversibility. First manufactured by Sony (Goodenough and Park, 2013) in 1991, LCO is the earliest commercialized cathode material. Although LCO has the advantages of high working voltage and good cycle performance, its anti-overcharge ability is poor. Moreover, the cobalt element resources are scarce, expensive, and toxic. There are still many challenges in the long-term cycle stability and safety. Sun et al. (2019) proposed a Ti-doped LCO cathode material, which suppressed the phase transition during cycling and increased the specific capacity to 205 mAh g^−1^ (4.5 V). The capacity retention rate was 97% after 200 cycles, and the performance was significantly enhanced. Zhang et al. (2019) et al. reported that a trace amount of Ti-Mg-Al co-doping synergistically promoted the cycling stability of LCO at 4.6 V. Xie et al. (2017) deposited LiAlO_2_ interfacial layers on LCO electrodes, which exhibited a reversible specific capacity close to 200 mAh g^−1^ at a high voltage of 4.6 V, and maintained a high capacity retention rate after 50 cycles. Lin et al. (2023) prepared core-shell structured LiCoO_2_ (CS-LCO) by a simple two-step multi-element co-doping strategy, with high-diffusivity Al^3+^/Mg^2+^ ions occupying the core and low-diffusivity Ti^4+^ ions enriching the shell. At a high cut-off voltage of 4.6 V, the single crystal CS-LCO maintained a reversible capacity of 159.8 mAh g^−1^ after 300 cycles, with a retention rate of nearly 89%.

In 1997, Padhi et al. first used LiFePO_4_ as the cathode material of lithium-ion batteries. It is green and environmentally friendly, safe and stable in structure, rich in raw materials, long in cycle life and low in price. Therefore, LFP was once considered the best choice for energy storage and new energy vehicle batteries. However, as cathode material, LFP has some problems such as low conductivity, low specific capacity, poor high current discharge performance, and low ion diffusivity, due to the structural defects of the material. Based on this, scholars have studied a series of modification methods, such as ion doping, surface coating (Wang et al., 2008; Zhang et al., 2020) and controlling the particle size of lithium iron phosphate. Li et al. (2024) introduced a gelatin-derived carbon network into the nanoscale LFP cathode, effectively facilitating the transfer of electrons and Li^+^. As a result, the cathode exhibited excellent rate capacity and good cycle performance (capacity retention of 80% after 500 cycles). Madram and Faraji (2017) synthesized Na^+^ and K^+^ co-doped LiFePO_4_/C composites at two discrete sites through a solid-state reaction route, which demonstrated higher electrochemical performance with higher capacity transfer and kinetics. Sun et al. (2019) employed xylitol-PVA calcination to form a material with a carbon layer uniformly wrapping the surface of LiFePO_4_ particles, and the material showed excellent performance in volume energy density, electrochemical performance, electronic conductivity and tap density.

Ternary material NCM was first reported by Liu et al., in 1999. The ternary material NCM has α-NaFeO_2_ type layered structure, which is beneficial to the de-intercalation of Li^+^. The content of Ni determines the charge and discharge capacity, Co is beneficial to improve the rate performance, and Mn mainly stabilizes the lattice structure of the material (Ellis et al., 2010). At present, since the Ni contents of the commercialized ternary cathode materials (mainly NCM111 and NCM523) are not high enough, they cannot provide high energy density to meet the needs of new energy vehicles. Therefore, scholars have turned their attention to high-nickel ternary cathode materials (Ni content, x ≥ 0.6) with higher specific capacity and lower cost in recent years, such as NCM811. Li et al. (2019) studied La and Al doped and coating modified NCM811 cathode materials, and found that the mismatch between the host phase and the surface layer was minimized by La_2_O_3_ coating, and the oxidation of the electrode reduced the Ni concentration gradient in the outer surface area. This material displayed enhanced rate capability, cycle life, and storage stability in air, with 80% capacity retention after 480 cycles at 10 C. Sun (Yanxia et al., 2020) et al. investigated the effect of Na and Mg co-doping on NCM811. The co-doping of Na and Mg improved the cycling reversibility of the material, reduced the resistance, and improved the electrochemical performance. Dixit et al. (2017) revealed that the stabilizing effect of Al was due to the strong Al-O ion covalent bonding of Al(s)-O(p) overlapping and a high degree of charge transfer from Al to oxygen by the first-principle DFT calculations. Meanwhile, Al increased the Li diffusion barrier near the doping site. Qiu et al. (2019) employed a protocol for incorporating Zr and F into NCM materials using conventional solid-state sintering techniques. This doping method alleviated the electrochemical polarization and significantly enhanced the structural stability, and the composite achieved a high capacity retention rate of 90.5% after 200 cycles at 1 C. Zhu et al. (2023) investigated the electrochemical properties of polycrystalline and monocrystal NCM811 materials, and the results showed that polycrystalline samples had higher discharge capacity and better rate performance than monocrystal samples, while monocrystal materials had better capacity retention and cycle stability. In addition, owing to the similar proportion of nickel as NCM811, LiNi_0.8_Co_0.15_Al_0.05_O_2_ (NCA) is seen as an alternative to provide high energy and power output for electric vehicles. However, the safety, cost and availability limit the wide application of NCA batteries.

Overall, the research on battery cathode materials focuses on the modification of materials, and the performance of materials are improved by doping and wrapping nowadays. However, there are few studies on the effect of cathode materials on the safety of complete batteries. This paper aims to experimentally investigate the thermal characteristics of LIBs with different cathode materials under extreme thermal runaway conditions and absolute thermal stability conditions to assess their risks.

## 2 Experimental

### 2.1 Battery sample

Four types of fresh lithium-ion batteries commercially available were selected in the experiments, including LCO, NCA, NCM811 and LFP batteries. The LCO and LFP batteries were provided by the Hvvea Amperex Co., Ltd. (China). The NCA and NCM811 batteries were manufactured by the Jiangsu Sunpower Co., Ltd. (China). The specifications are listed in Table 1, but other precise chemical composition of these LIBs is still confidential from the manufacturers for now.

**TABLE 1:** Summary of 18,650 battery specifications.

Cathode (active material: conductive agent: binder, wt%)	Anode (active material: conductive agent: binder, wt%)	Electrolyte	Separator	Nominal voltage/V	Rated capacity/Ah	Internal resistance/mΩ	Cell design
LCO (95.9:1.2:2.9)	Graphite (96.8:1.2:2)	1 mol/L LiPF_6_/EC:DMC:EMC (10:80:10, vol%), 3% FEC (wt%)	PP	3.7	2.2	≤60	N_t_: 1 × 1
							N_w_: 19
							T_c_: 134 μm
							T_a_: 146 μm
NCA (98:1:1)	Graphite (96.8:0.2:3)	1.02 mol/L LiPF_6_/EC:PC:DEC (1:1:1, vol%)	PE	3.7	3.0	≤18	N_t_: 1 × 1
							N_w_: 24
							T_c_: 114 μm
							T_a_: 98 μm
NCM811 (96:2:2)	Graphite (95:2:3)	1.1 mol/L LiPF_6_ +0.8 mol/L LiBOB/EC:EMC:DMC (25:40:35, vol%), 2.3% VC (wt%), 1.3% PS (wt%), 2% ES (wt%)	PE	3.7	2.5	≤18	N_t_: 1 × 1
							N_w_: 26
							T_c_: 90 μm
							T_a_: 102 μm
LFP (95.5:2:2.5)	Graphite (96:0.8:3.2)	1 mol/L LiPF_6_/EC:DMC:EMC (1:1:1, vol%)	PE	3.2	2.0	≤25	N_t_: 1 × 1
							N_w_: 24
							T_c_: 135 μm
							T_a_: 90 μm

Note: N_t_, number of tabs; N_w_, number of windings; T_c_, thickness of the cathode layer; T_a_, thickness of the anode layer.

### 2.2 Accelerating rate calorimeter (ARC) test

**FIGURE 1:**
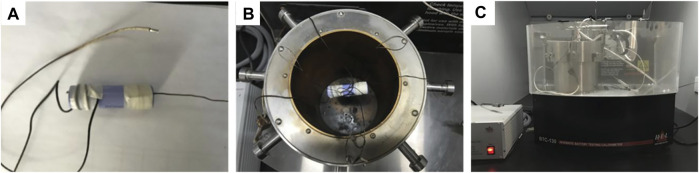
Experiment device of the ARC tests: **(A)** the heating wire and temperature sensor were glued to the battery, **(B)** the battery at 100% SOC was fixed inside the calorimetric chamber center, **(C)** the ARC made by Thermal Hazard Technology (THT).

Using ARC made by the Thermal Hazard Technology (THT), the thermal characteristics of the exothermic reaction process can be simulated when the internal heat of the battery cannot be dissipated in time, making the experiment closer to the real reaction process. The temperature measuring thermocouple was fixed on the surface of the battery, and the battery at 100% SOC was fixed inside the calorimetric chamber (Figure 1). The ARC tests were performed in heat-wait-search mode from 60°C to 300°C to detect the heat release of the battery sample. Heating was operated in 5°C steps, so that the onset of critical thermal events would not be missed. And the followed 15-min wait time was intended to make the sample, the sample container and the calorimetric chamber reach thermal equilibrium, so that the system could more accurately search for the self-exothermic heat of the sample. When the temperature rise rate exceeded 0.03°C min^−1^, the sample was considered to be self-heating, and the ARC was switched to adiabatic mode and parameters such as the sample heat release rate were recorded.

### 2.3 Isothermal battery testing calorimeter (iso-BTC) test

**FIGURE 2:**
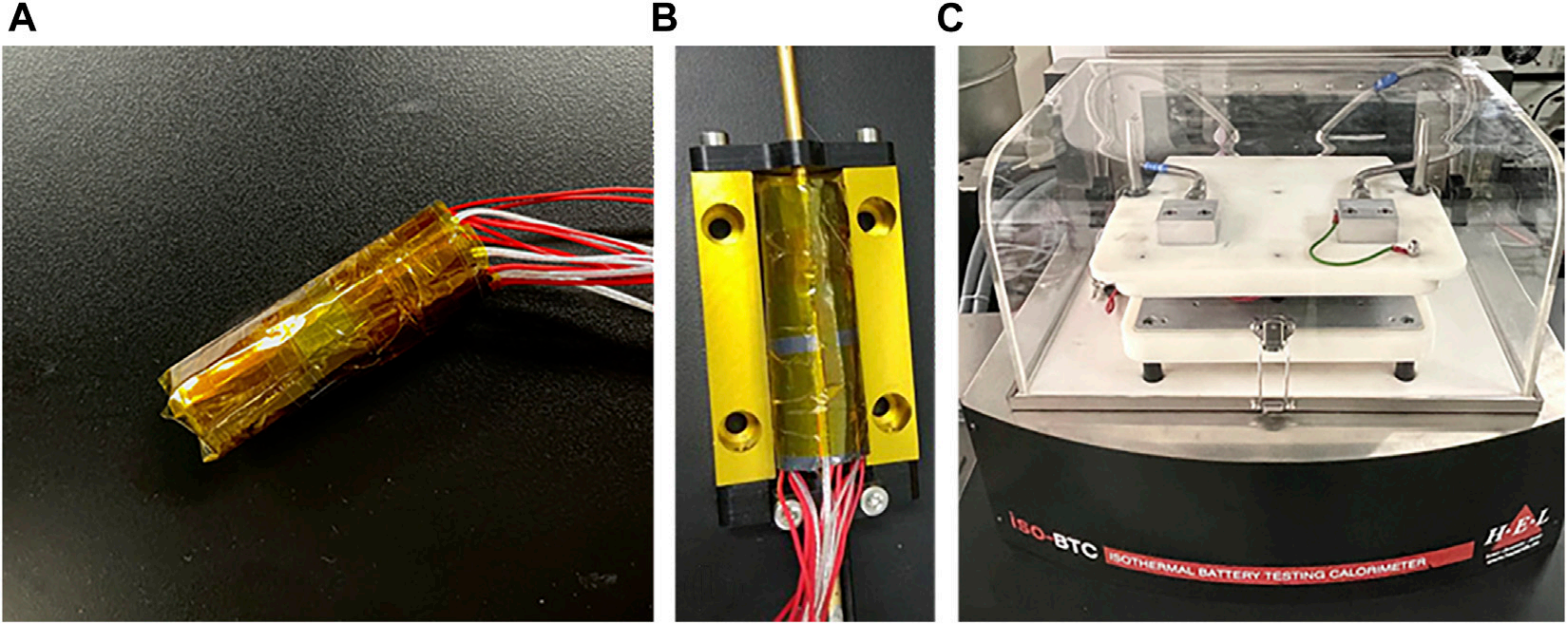
Experiment device of the iso-BTC tests: **(A)** power compensators and temperature sensors were arranged on the battery surface, **(B)** the battery tightly wrapped by the special thermally conductive graphite paper was put into the test board center, **(C)** the iso-BTC made by Hazard Evaluation Laboratory.

The experimental equipment for lithium-ion battery charge-discharge thermal characteristics tests consists of iso-BTC, Huber circulator and charge-discharge instrument (20 V-10 A). The iso-BTC made by the Hazard Evaluation Laboratory (London, United Kingdom) can monitor the real-time thermal characteristics of the battery based on the principle of power compensation. Prior to the test, the battery sample was discharged to its discharge cut-off voltage according to its standard charge-discharge procedure. Two power compensators and two temperature sensors were arranged at the dispersed positions of the positive and negative electrodes of the battery. After tightly wrapped by the special thermally conductive graphite paper and inserted into the professional adapter, the battery was put into the center of the test board (Figure 2). In order to ensure the consistency of the experiment, the battery charging process employed a constant current of 0.5 C to the charging cut-off voltage, and then the battery was charged with a constant voltage until the current was less than 0.02 C. The battery discharging process employed a 0.5 C constant current until the discharge cut-off voltage was reached. The heat release rate, energy release, current, voltage and temperature of the battery sample were monitored in real time during the whole charging and discharging processes.

## 3 Results and discussion

### 3.1 Battery performance under extreme thermal runaway conditions

**FIGURE 3:**
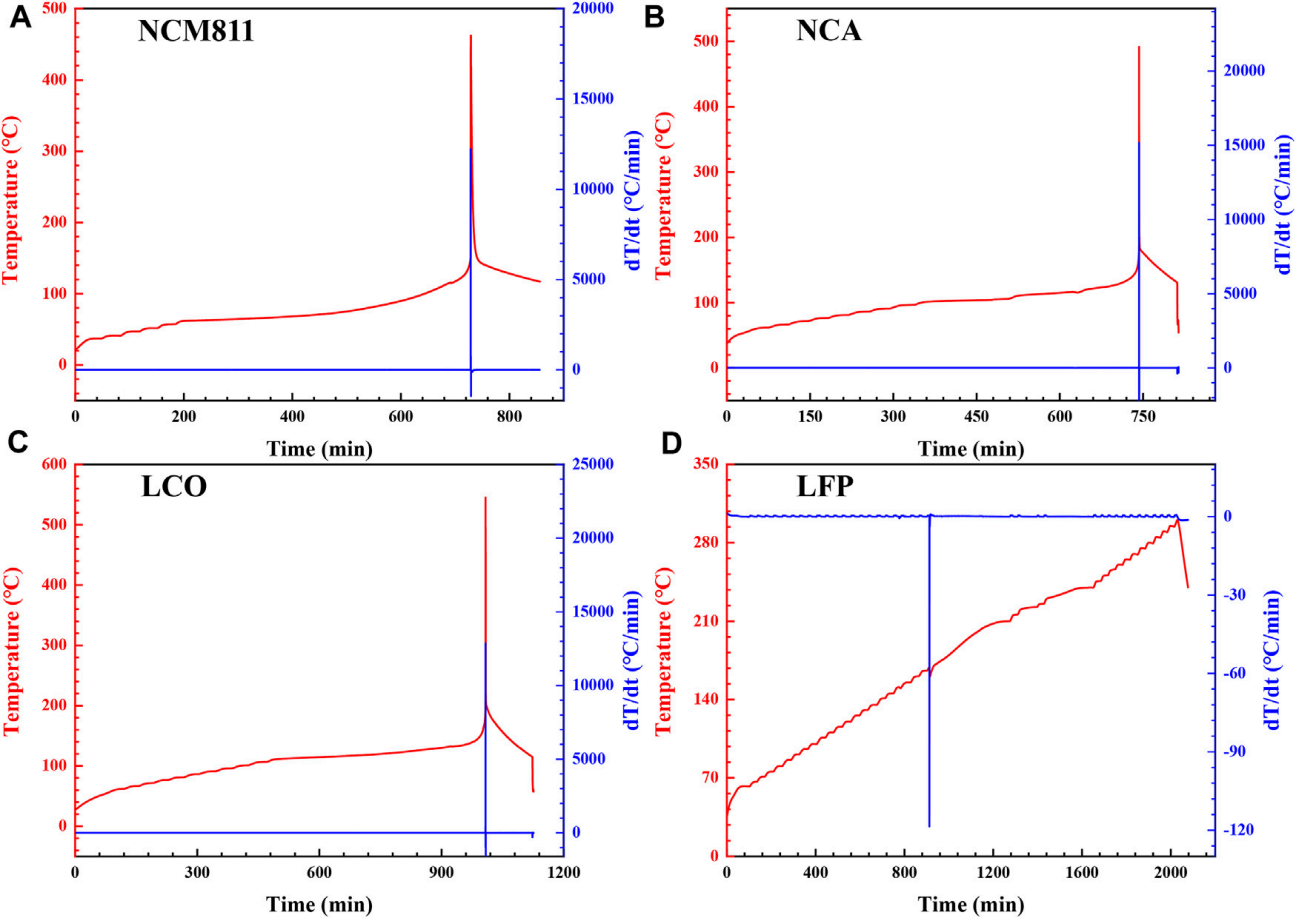
Thermal runaway plots of 18,650 LIBs: **(A)** NCM811; **(B)** NCA; **(C)** LCO; **(D)** LFP.

Reactions involving multiple internal components may occur simultaneously when a lithium-ion battery develops thermal runaway, rather than in a hypothetical sequence. Therefore, the ARC experiment of the whole battery is the necessary test to explore the real situation and the complete process of thermal runaway of commercial batteries, and the test results are shown in Figure 3. The combination of three temperatures, (T_oer_, T_tr_, T_max_), is employed as the characteristic temperature of the key thermal runaway features (Duh et al., 2021), as listed in Table 2. The onset temperature of exothermic reactions (T_oer_) is defined by a Self-Heating Rate (SHR) of SHR >0.03°C min^-1^, which represents the overall thermal stability of a battery. The thermal runaway temperature (T_tr_) itself is quantitatively defined as SHR first surpasses 10°C min^−1^, which can be seen as the critical point separating the temperature mild increase and sharp rise (Friesen et al., 2016; Galushkin et al., 2018). T_max_ is the maximum temperature that the batteries can reach during thermal runaway. Since cathode decay energetics can be estimated from the maximum Heating-Rate (HR) in the ARC, HR_max, nominal_ is found using the nominal capacity of each battery (2.5 Ah for NCM811, 3 Ah for NCA, 2.2 Ah for LCO, and 2 Ah for LFP) on the basis of HR_max_ to be normalized for the comparison of different batteries (Barkholtz et al., 2019).

**TABLE 2:** Thermal runaway data of four different 18,650 LIBs.

Sample	T_oer_ (°C)	T_tr_ (°C)	T_max_ (°C)	HR_max_ (°C min^-1^)	HR_max, nominal_ (°C min^-1^ Ah^-1^)
NCM811	62.49	147.35	462.52	12,218.52	4,887.41
NCA	101.71	165.44	491.84	14,634.44	4,878.15
LCO	111.78	180.16	545.11	12,850.60	5,841.18
LFP	62.50	-	239.26	0.36	0.18

As can be seen from Figure 3 and Table 2, the T_oer_ of NCM811, NCA, LCO and LFP as low as 62.49°C, 101.71°C, 111.78°C and 62.50°C are detected, respectively, originating from the solid electrolyte interface (SEI) decomposition. For commercial batteries with carbon-based anodes, the above results are coincided with statistical data of T_oer_ (60°C–120°C), which are mainly determined by the composition of the electrolyte and the resulting SEI layer. The T_tr_ observed at 147.35°C, 165.44°C, and 180.16°C for NCM811, NCA and LCO, respectively, is closely related to the separator material of the battery. The main heat source of the battery between T_oer_ and T_tr_ is the exothermic reaction inside the anode, while the heat originated from the cathode is negligible. The surface of the graphite is usually covered with a continuous SEI film, which begins to crack when the temperature rises to about T_oer_. The decomposed SEI film cannot prevent the embedded lithium from coming into contact with the electrolyte, which react with each other to produce significant heat. This reaction is similar to the “formation” process in battery manufacturing. The SEI layer regenerates during this process, but is poor in density and poor in ability to prevent further reaction. Therefore, the thermal generation of the anode will continue until the battery is heated to T_tr_. At present, the commonly used substrates for commercially available lithium-ion battery separator are PE (polyethylene) and PP (polypropylene). When the temperature reaches its melting point (e.g., 130°C for PE based separator, or 170°C for PP based separator), the PE/PP-based diaphragm melts and contracts. As the area of the separator shrinks, the anode and cathode electrodes gradually lose isolation. Once the anode and cathode electrodes come into contact, the internal-short-circuit (ISC) occurs, rapidly releasing the electrical energy stored in the battery and triggering the generation of a large amount of heat. Since the redox reaction does not occur until temperatures of 230°C or higher, the ISC is responsible for heating the battery sample from T_tr_ to 230°C. That is to say, ISC is a key trigger for thermal runaway, but not the main heat source for thermal runaway. The major heat source that causes the temperature to rise sharply from T_tr_ to T_max_ is the redox reaction of the anode and cathode electrodes at higher temperature (Feng et al., 2019).

The T_max_ that the batteries can reach during thermal runaway for NCM811, NCA, LCO and LFP are observed at 462.52°C, 491.84°C, 545.11°C and 239.26°C, and the HR_max, nominal_ are determined to be 4,887.41°C min^−1^ Ah^−1^, 4,878.15°C min^−1^ Ah^−1^, 5,841.18°C min^−1^ Ah^−1^ and 0.18°C min^−1^ Ah^−1^, indicating that the LFP produces minimal thermal runaway consequence owing to the high strength of phosphorus oxygen bond in phosphate group and the lowest reaction heat release. The T_max_ at 239.26°C for LFP is much lower than the auto-ignition temperature (AIT) of organic carbonate, thus there is no thermal runaway combustion or spontaneous combustion when the LFP battery ruptures. As for other non-LFP batteries, their fire hazard (T_max_ > AIT of diethyl carbonate, 445°C) and rate hazard (high HR_max, nominal_) are all unacceptable, and the thermal runaway danger level of these 18,650 LIBs can be ranked as LCO > NCA > NCM811 >> LFP, by judging from the values of T_max_ and HR_max, nominal_. When the cathode material is charged, the low valence Ni^2+^ and Co^3+^ will be oxidized into high valence Ni^3+^, Ni^4+^ and Co^4+^. These high valence ions will get electrons and turn into low valence states when heated, while active oxygen ions, such as O^2-^, O^−^, O_2_^2-^, will lose electrons to form oxygen release, further oxidizing the electrolyte to generate a lot of heat. Therefore, the high valence ion is an important factor to reduce the thermal stability of the positive electrode material. The higher the nickel and cobalt content, the higher the lithium-ion battery capacity, but the worse the thermal stability.

**FIGURE 4:**
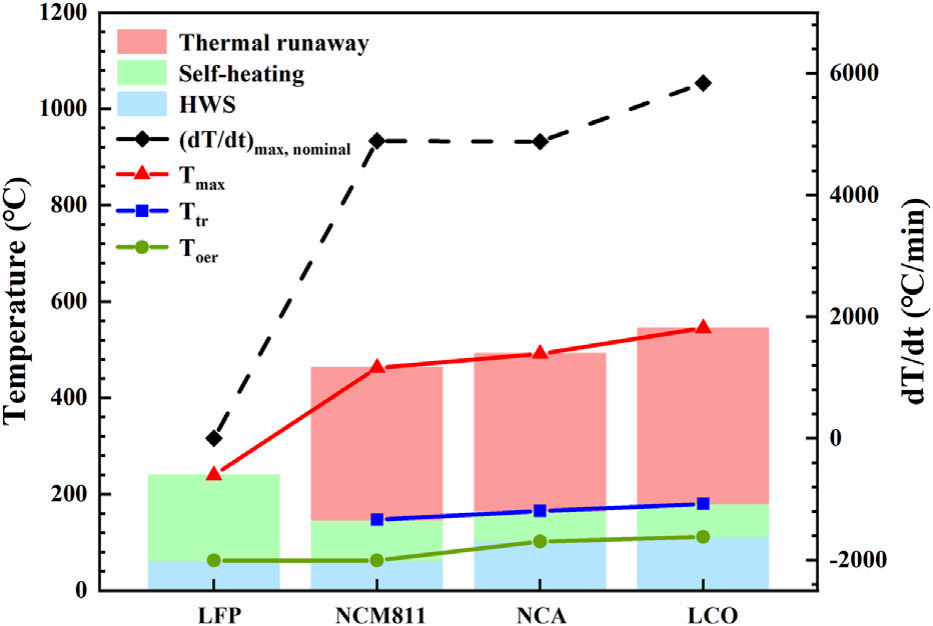
Three stages of thermal characterization for different 18,650 LIBs.

Figure 4 compares the average value of thermal runaway characteristic parameters in three repeated experiments of each battery, and the process of self-heating reaction to thermal runaway is clearly divided into three stages. Stage I (blue part) is the thermal stabilization stage, and the temperature step corresponds to the heat-wait-search (HWS) operating mode of ARC. Once the battery temperature reaches T_oer_, that is, ARC detects the self-heating reaction of the battery, ARC begins to track the temperature change of the battery, forming adiabatic conditions and entering the stage II (green part). T_tr_ is the end temperature of stage II, and then the temperature rises sharply, entering the thermal runaway stage (stage III, red part), in which the lithium-ion battery is at risk of fire and explosion at any time. Depending on the length of the red and green segments, the effects of different cathode materials on the thermal safety of lithium-ion batteries can be visually compared. The longer the red segment and the shorter the green segment, the worse the thermal safety of the battery. The T_max_ and HR_max, nominal_ of LCO battery are the highest, and the red segment is the longest, indicating that its thermal runaway consequence is the most serious. Secondly, compared with NCM811, NCA has a shorter green segment and a longer red segment, indicating a higher risk of thermal runaway. The T_tr_ of LFP is not even detected, revealing that the LFP is the safest of the four batteries.

### 3.2 Battery performance under absolute thermal stability conditions

**FIGURE 5:**
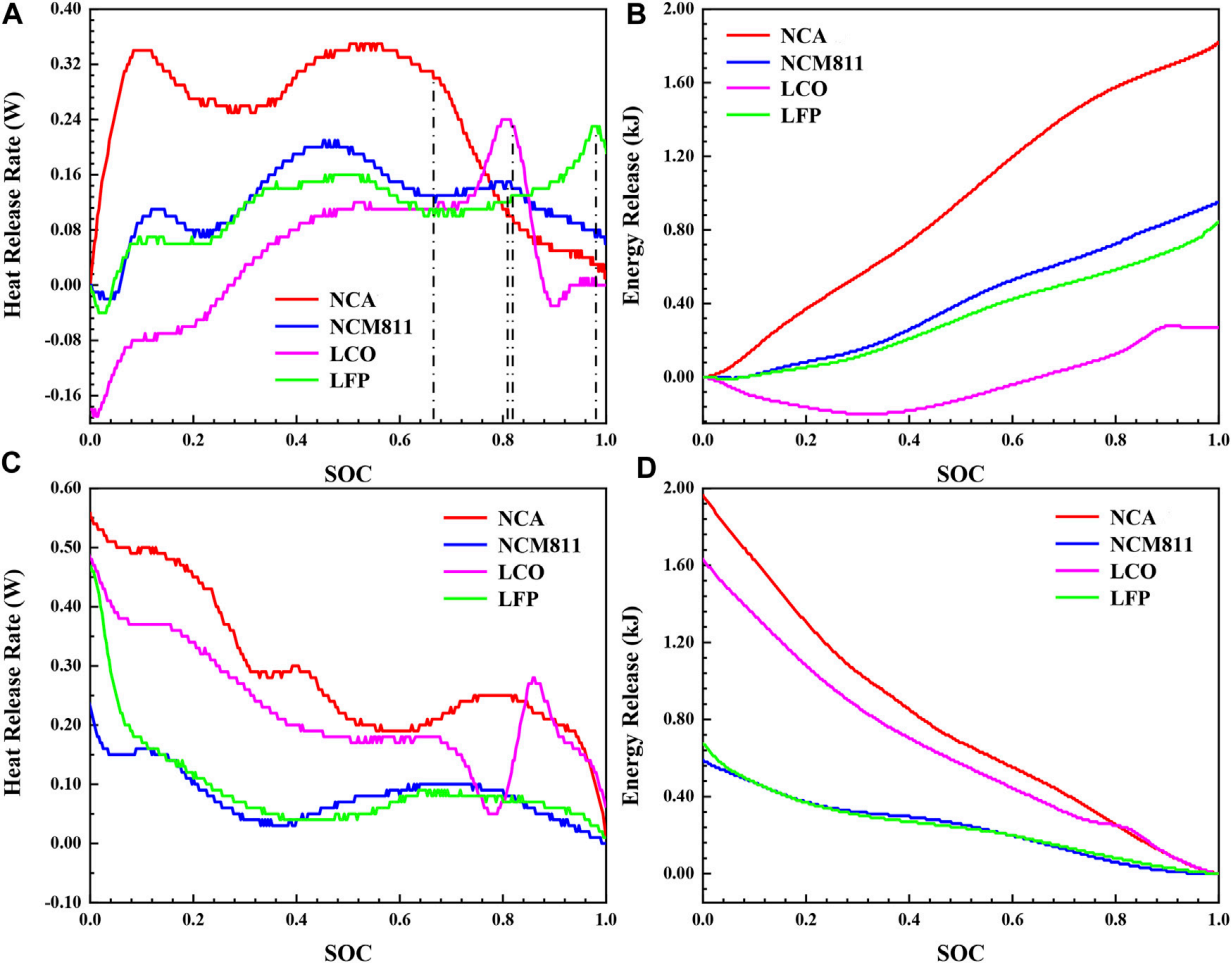
The thermal characteristics during charging and discharging process of four different 18,650 LIBs at 30°C: **(A)** heat release rate and **(B)** energy release during charging process, **(C)** heat release rate and **(D)** energy release during discharging process.

The safety risks of the lithium-ion battery are mainly reflected in two aspects. First, the thermal runaway problem caused by extreme conditions such as acupuncture, extrusion, and heat source. The second is the accumulation of heat generated by the normal charging and discharging process. The changes of heat release rate and energy release with SOC in the charging and discharging process of four lithium-ion batteries with different cathode materials at 30°C were compared by isothermal calorimetry tests to analyze the heat generation characteristics of the four commercial batteries during normal working process, and the results were shown in Figure 5. According to the battery heat generation rate model established by Bemadi D, the heat generation can be divided into two parts (Eqs 1–3). One is reversible heat (Q_rev_), which is the entropic heat produced by the electrochemical reaction inside the battery. The other is the irreversible heat (Q_irre_) generated by the internal resistance of the battery, which is divided into polarization heat and ohmic heat.

$$Q=Q_{rev}+Q_{irre}$$ (1)

$$Q_{rev}=-I\left(T\frac{\partial U}{\partial T}\right)t$$ (2)

$$Q_{irre}=I\left(U-V\right)t=I^{2}RT$$ (3)

The constant current charging stage and constant voltage charging stage have been distinguished by dash lines in Figure 5A. Compared with the constant current charging stage, the heat release rate of the battery in the constant voltage charging stage decreases rapidly and the growth rate of energy release slows down, due to the current decreases sharply after entering the constant voltage charging process. Therefore, the constant current process is the main stage of heat accumulation in the standard charging process. As shown in Figure 5, the heat generation changes in the constant current charging and discharging process present symmetrical upward and downward trends on the whole. The reason for this phenomenon is that the reversible heat caused by the entropy change of the electrode material exhibits an almost transversely zygomorphic distribution, and in contrast, the ohmic heat and polarization heat remain basically constant at the small charge-discharge rate of 0.5 C. Part of the left and right dislocation is due to the fact that the thermocouple monitors the battery surface temperature during the test, and there is a lag in the response to the battery heat generation, resulting in the right shift of the heat generation rate curve during the charging process and the left shift during the discharging process. Therefore, it is assumed that the Q_irre_ in the charge and discharge process is the same (Mao et al., 2021), which is the symmetry axis of the charge and discharge energy release curves in Figures 5B, D. Q_rev_ can be calculated according to the heat flow difference of charge and discharge process, as shown in Eq. 4 and Eq. 5. The calculation results are shown in Table 3.

$$Q_{rev}=\frac{Q_{dis}-Q_{cha}}{2}$$ (4)

$$Q_{irre}=\frac{Q_{dis}+Q_{cha}}{2}$$ (5)

**TABLE 3:** The reversible heat and irreversible heat of various 18,650 LIBs at 30°C.

Sample	Q_cha_ (kJ)	Q_dis_ (kJ)	Q_total_ (kJ)	Q_irre_ (kJ)	Q_rev_ (kJ)	
					Charge	Discharge
NCA	1.82	1.98	3.80	1.90	−0.08	0.08
NCM811	0.95	0.59	1.54	0.77	0.18	−0.18
LCO	0.27	1.63	1.90	0.95	−0.68	0.68
LFP	0.85	0.67	1.52	0.76	0.09	−0.09

The Q_total_ of NCA (3.80 kJ) is the largest in the complete standard charge and discharge process, which is 2.5 times of the Q_total_ of LFP (1.52 kJ). The reason is that the capacity of NCA (3 Ah) is significantly higher than that of the other three batteries, and the charging and discharging current is the largest, resulting in a remarkable increase in Q_irre_ proportioned to the square of the current. In the process of constant current charging, the heat release rate of NCA is always the largest, and that of the LCO is relatively minimum overall. The fluctuation of heat release rate is mainly affected by the entropy heat coefficient (∂U/∂T). The entropy heat coefficient of the battery at different state of charge (SOC) is related to the phase transformation of the cathode material, the structural transformation of the anode material and lithium deintercalation reaction. Obviously, there is a peak value at the main reaction interval (20%–60%) of lithium deintercalation in the heat release rate curve of the four batteries (Figure 5A), indicating that the entropy change of the battery in this interval is the largest. Until the end of charging, the energy release of LCO (0.27 kJ) is only 14.84% of that of NCA (1.82 kJ), due to the reversible thermal effect of LCO shows a more significant endothermic effect. The reversible phase transition between the hexagonal phase and the monoclinic phase of LCO brings about a large entropic heat coefficient. In the discharge process, the heat generation of NCA is still the largest. It is worth noting that the heat generation of LCO increases significantly to exceed NCM811 and LFP during the discharge process, owing to the more conspicuous exothermic effect of the reversible reaction of LCO. In summary, NCA generates more heat under the same charge-discharge rate, which is more likely to cause battery safety problems. For LCO, more attention should be paid to its thermal management during discharge process.

**FIGURE 6:**
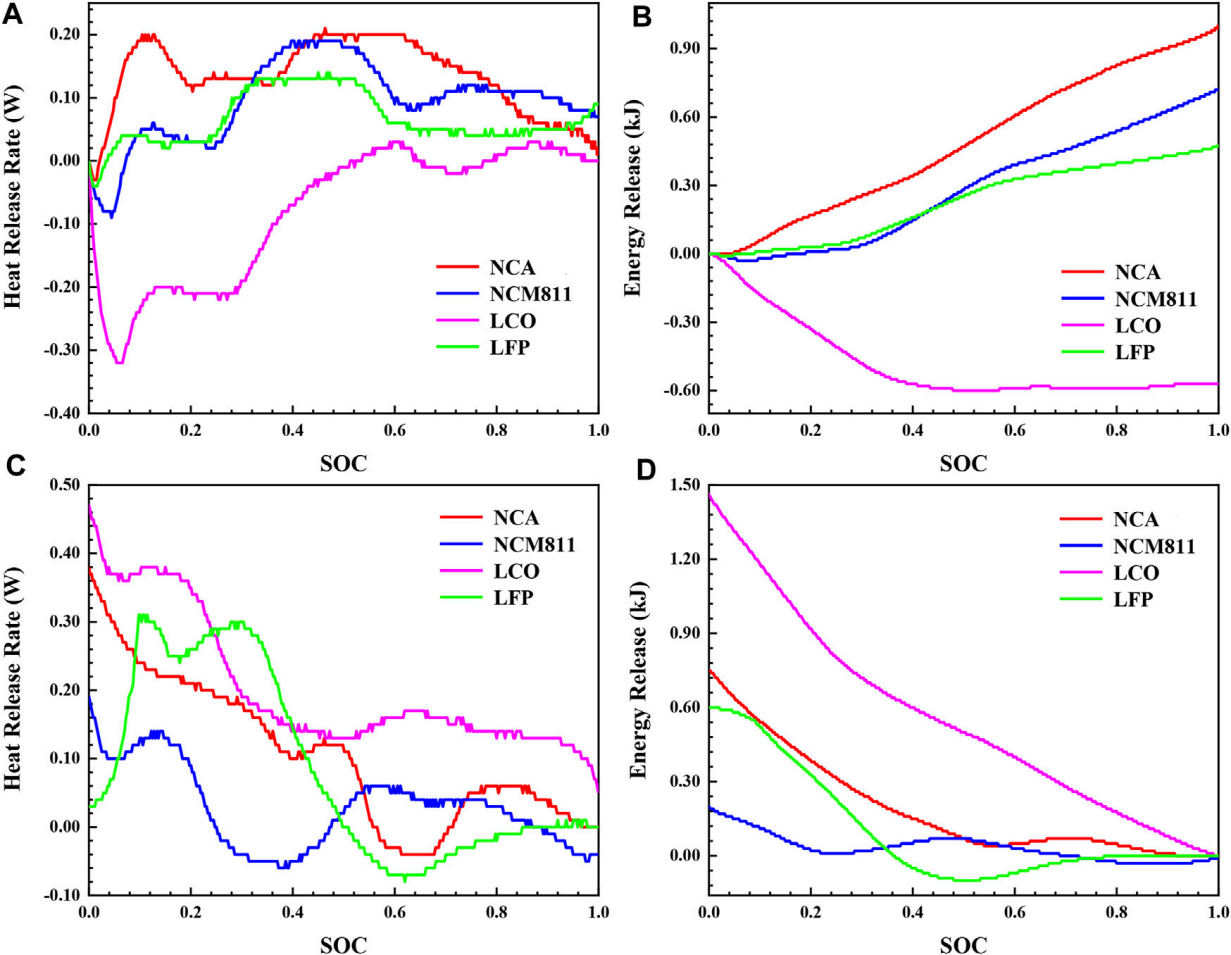
The thermal characteristics during charging and discharging process of four different 18,650 LIBs at 60°C: **(A)** heat release rate and **(B)** energy release during charging process, **(C)** heat release rate and **(D)** energy release during discharging process.

In order to investigate the influence of ambient temperature on the thermal behavior of batteries with different cathode materials during normal charging and discharging process, isothermal calorimetry tests were also carried out at 60°C as a supplementary comparison. The results are shown in Figure 6. 30°C in Figure 5 is the normal environmental condition of the actual working situation of the lithium-ion battery, and 60°C is the case of not cooling in summer. The comparison between Figure 5 and Figure 6 shows that the ambient temperature has a significant effect on the thermal behavior of the battery. When the ambient temperature is high, the heat release rate curve shifts downward as a whole, and the energy release decreases as well (Table 4). Most notably, Q_total_ of NCA reduces by 53.95%, and the Q_irre_ reduces by 53.68%. This phenomenon shows that the main reason for the reduction of heat generation is that when the temperature rises, the diffusion rate of lithium ions becomes faster, and the electrochemical reaction rate increases, resulting in a decrease in the internal resistance of the battery.

**TABLE 4:** The reversible heat and irreversible heat of various 18,650 LIBs at 60°C.

Sample	Q_cha_ (kJ)	Q_dis_ (kJ)	Q_total_ (kJ)	Q_irre_ (kJ)	Q_rev_ (kJ)	
					Charge	Discharge
NCA	1.00	0.75	1.75	0.88	0.12	−0.13
NCM811	0.72	0.20	0.9	0.46	0.26	−0.26
LCO	−0.57	1.46	0.89	0.45	−1.02	1.01
LFP	0.48	0.60	1.08	0.54	−0.06	0.06

## 4 Conclusion

In this paper, the thermal safety of lithium-ion batteries with different cathode materials (NCA, NCM811, LCO and LFP) was compared under adiabatic and isothermal conditions by ARC and iso-BTC. The T_max_ for NCM811, NCA, LCO and LFP are observed at 462.52°C, 491.84°C, 545.11°C and 239.26°C, and the HR_max, nominal_ are determined to be 4,887.41°C min^−1^ Ah^−1^, 4,878.15°C min^−1^ Ah^−1^, 5,841.18°C min^−1^ Ah^−1^ and 0.18°C min^−1^ Ah^−1^, indicating that the LFP produces minimal thermal runaway consequences owing to the high strength of phosphorus oxygen bond in phosphate group and the lowest reaction heat release. As for other non-LFP batteries, their fire hazard and rate hazard are all unacceptable, and the thermal runaway danger level can be ranked as LCO > NCA > NCM811 >> LFP. The high valence ion is an important factor to reduce the thermal stability of the cathode material. The higher the nickel and cobalt content, the higher the lithium-ion battery capacity, but the worse the thermal stability.

The Q_total_ of NCA is the largest in the complete standard charge and discharge process, which is more likely to cause battery safety problems. The reason is that the capacity of NCA is significantly higher than that of the other three batteries, and the charging and discharging current is the largest, resulting in a remarkable increase in Q_irre_ proportioned to the square of the current. And for LCO, more attention should be paid to its thermal management during discharge process. When the ambient temperature rises, the energy release decreases, due to the decrease in the internal resistance of the battery caused by the increase of the diffusion rate of lithium ions and the electrochemical reaction rate. These studies are expected to have important implications for the subsequent safe design of commercial lithium-ion batteries with different cathode materials.

## Data Availability

The original contributions presented in the study are included in the article/Supplementary material, further inquiries can be directed to the corresponding authors.
